# Patient safety topics, especially the second victim phenomenon, are neglected in undergraduate medical and nursing curricula in Europe: an online observational study

**DOI:** 10.1186/s12912-023-01448-w

**Published:** 2023-08-24

**Authors:** Alicia Sánchez-García, Pedro José Saurín-Morán, Irene Carrillo, Susanna Tella, Kaja Põlluste, Einav Srulovici, Sandra C Buttigieg, José Joaquín Mira

**Affiliations:** 1 Foundation for the Promotion of Health and Biomedical Research of the Valencia Region, FISABIO, Alicante, Spain; 2 Health Psychology Department, Miguel Hernandez University, Elche, 03202 Spain; 3 Faculty of Social Services and Health Care, LAB University of Applied Sciences, Lappeenranta, 53850 Finland; 4 Institute of Clinical Medicine, University of Tartu, Tartu, Estonia; 5 Cheryl Spencer Department of Nursing, University of Haifa, Haifa, 3498838 Israel; 6 Department of Health Systems Management and Leadership, Faculty of Health Sciences, University of Malta, Msida, 2080 MSD Malta

**Keywords:** Adverse events, Interprofessional communication, Patient safety, Quality of care, Risk management, Safe practices, Second victims

## Abstract

This study aims to assess the inclusion of second victims and other patient safety issues in the curricula of undergraduate medical and nursing degrees in the countries participating in the European Researchers’ Network Working on Second Victims (The ERSNT Consortium, COST Action 19,113). A review of medical and nursing school curricula in 206 universities was carried out, using their websites to search for subjects addressing “patient safety”, “quality of care”, “risk management”, “safe practices”, “interprofessional communication”, “adverse events”, and “second victims”. There was substantial variability in the extent of training for patient safety. Forty-four out of 88 nursing schools and 74 of 118 medical schools did not include any of the patient safety topics studied. The most frequent in both nursing and medicine was “interprofessional communication”, followed by “quality of care” and basic aspects on “patient safety”. The second victim phenomenon was present in only one curriculum of the total sample. Our study showed that patient safety, especially the second victim phenomenon, is still neglected in medical and nursing curricula in European universities, although positive initiatives were also found. Given the frequency with which adverse events occur in health centres and the need to prepare students to deal with them adequately, additional efforts are needed to introduce patient safety elements into medical and nursing education.

**Supplementary Information:**

The online version contains supplementary material available at 10.1186/s12912-023-01448-w.

## Background

Patient safety has been defined as “a framework of organized activities that creates cultures, processes, procedures, behaviours, technologies and environments in health care that consistently and sustainably lower risks, reduce the occurrence of avoidable harm, make error less likely and reduce impact of harm when it does occur” [1]. Nowadays, patient safety is considered an area of special relevance for clinical practice. Since the Institute of Medicine (IOM) report *To Err is Human* highlighted the importance of preventing errors in healthcare, there has been a global movement for patient safety along with a boom in scientific literature around the concept and principles [2].

Health professionals are expected to know how to deal with the inherent risks of clinical practice, incorporate a quality and patient safety perspective into their work, and minimise the risk of adverse events. However, clinicians cannot achieve these objectives if they are not properly trained, so patient safety needs to be integrated into health education curricula from the earliest years so that students can assimilate the concepts and skills of patient safety and apply them to their future clinical practice.

A multitude of national and international health institutions, including the World Health Organization (WHO), have published recommendations on including patient safety in the existing curricula of all healthcare professions [3–8]. Despite this guidance, different authors have found that many medical and nursing schools have not yet formally incorporated these contents into their curricula [1, 9–12]. In light of these reports, it is necessary to know the current state of play in terms of incorporating patient safety aspects into healthcare curricula, to ensure that professionals are equipped to fulfil their expected role in improving patient safety.

Thus, this study aims to assess the inclusion of subjects related to patient safety and quality of care, as well as the phenomenon of second victims, in the undergraduate medical and nursing school curricula of public and private universities in Europe.

## Methods

This observational study was conducted on a random selection of university websites in the European countries participating in the ERNST Consortium (The European Researchers’ Network working on Second Victims, COST Action 19113), a European consortium, funded by COST (European Cooperation in Science and Technology), focused on the study of the impact of the second victim phenomenon on patient safety. The countries participating in the ERSNT Consortium in that period were: Austria, Belgium, Bosnia and Herzegovina, Croatia, Czech Republic, Denmark, Estonia, Finland, France, Germany, Iceland, Ireland, Israel, Italy, Lithuania, Malta, Moldova, Netherlands, North Macedonia, Norway, Poland, Portugal, Romania, Serbia, Slovakia, Spain, Sweden, Switzerland, and Turkey. Universities were randomly selected from a list of universities teaching medicine and nursing identified by using different sources available online. In case of doubt, we consulted with partners in the European Researchers’ Network working on Second Victims countries. A maximum of 15 universities were included in the cases of countries with many medical or nursing schools. Initially, two independent reviewers selected 5 universities per country (except for those countries that did not have 5 universities with medicine and nursing degrees) and applied the inclusion and exclusion criteria. For this first selection, the random function of a spreadsheet with the list of universities was used. Specifically, they first checked whether the university offered the nursing/medicine degree or both, and then whether the curriculum and course details were publicly available, as lack of access to this information was an exclusion criterion. Nevertheless, this criterion was not fully applied in the case of small countries with less than 5 medical and nursing universities. In general, universities that did not publish the curricula or module descriptions on their websites were excluded from the analysis. Because some countries had very few universities (< 5) awarding medical or nursing degrees, if a list of modules was available, the university was included, even when the module description was not detailed. This first review noted the expectation of a low presence of curricular content specifically referring to patient safety, second victims and quality of care. Consequently, and with the intention of avoiding the under-representation of universities from countries with a higher number of higher education centres and the over-representation of those with fewer than five universities, we decided to make a second random selection from the list of universities not yet screened, up to a maximum of 5 per country that met the inclusion criterion regarding curricular content. Publicly available curricula were searched for subjects related to quality of care and patient safety, including risk management, safe practices, interprofessional communication, open disclosure, and second victims.

Altogether, the screening of universities websites in 29 European countries was carried out from 18 July to 25 August 2022. A total of 88 universities and colleges offering nursing degrees were initially identified, along with 118 universities offering medical degrees. In nursing, only bachelor’s degrees were included; however, in medicine some universities divide compulsory education into bachelor’s (3 years) and master’s (minimum 3 years) programmes, so graduate-level curricula had to be included in these cases.

University websites were reviewed in the national language and in English whenever an English version was available. Where only the national language was available, Google Translate service was used. The analysis included medical and nursing schools using the following terms in the course curriculum or module descriptions: “patient safety”, “quality of care”, “risk management”, “safe practices”, “interprofessional communication”, “adverse events”, and “second victims”. Curricula and modules where the terms were present were included, but also those that referred to the concept, following WHO conceptualisation framework for patient safety [13]. Decisions on whether a particular concept was related to patient safety were based on researchers’ best judgement. Because universities with the exact terms were included, some universities had to be excluded in the last review because the terms searched did not refer to the established definitions. For example, modules and curricula with a description of basic communication skills (“communicative styles”, “listen attentively and allow pauses in conversation”) or specific situations not related to patient safety (“communication and relationships at the end of life”, “communication for researchers”) were excluded. Communication was likewise not included when it referred to linguistic competencies for foreign students. On the other hand, contents such as “guarantee the quality of communication between professionals in patient transfers”, “informing the patient of adverse events”, and “acquiring knowledge about the need for adequate communication with patients, other health professionals, and the non-health sector” were included. The entire process of searching, screening and extracting information on curricula was carried out independently by two reviewers. In case of discrepancy, the judgement of a third reviewer was applied.

The following data were collected from the selected universities:

- Name of the university.
- Type of university (public or private).
- Topics related to any of the terms and link to the module plan, if available.
- Module credits.
- Compulsory versus elective nature of the module.
- Academic year and semester.
- Information about the module: contents, objectives, and topics.
- Link to the curricula.

The final sample of nursing and medical schools, both public and private, consisted of 44 university nursing degree programmes and 44 university medical degree programmes (see additional file 1 for excluded and included universities and curricula).

**Fig. 1:**
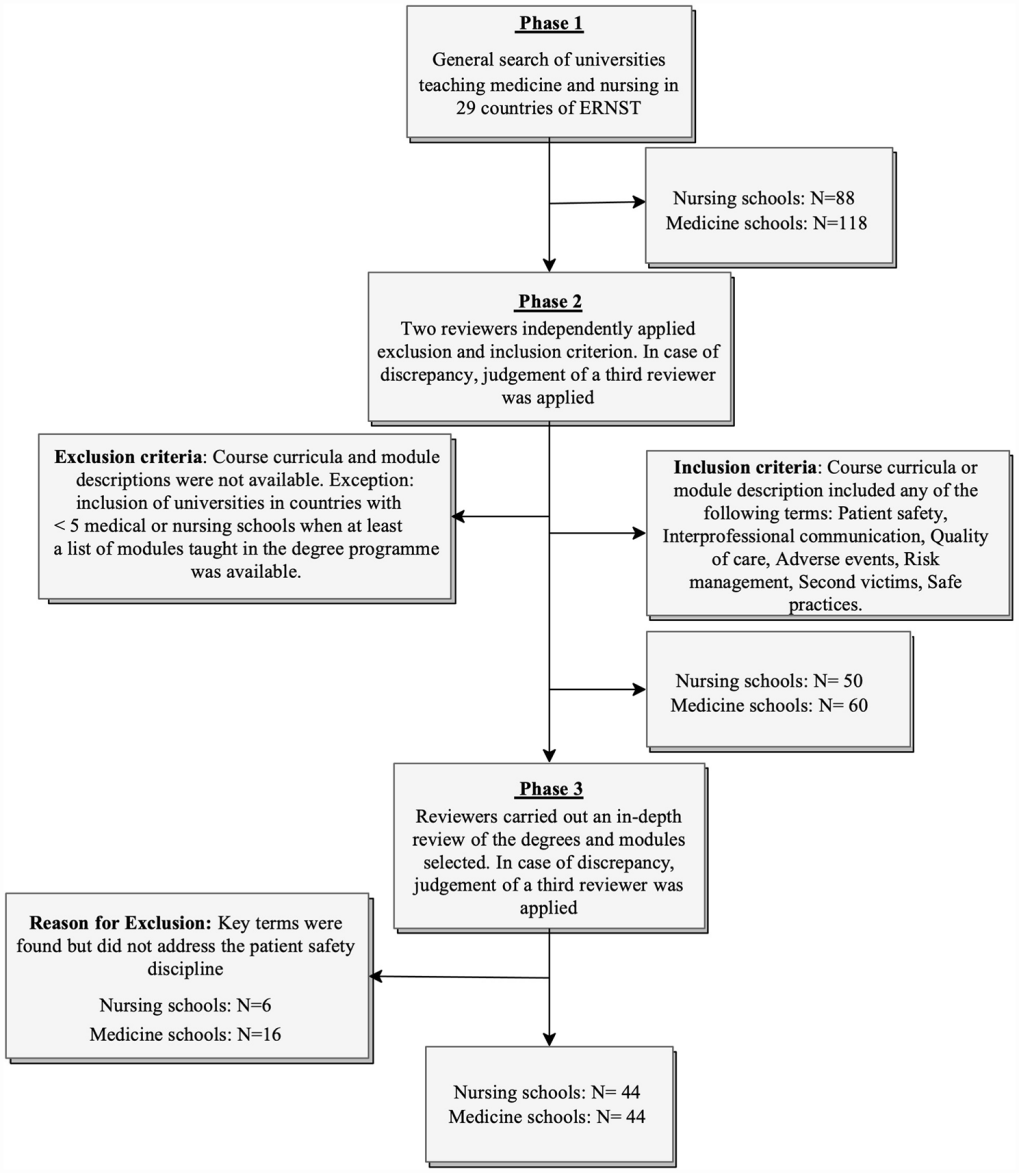
Flow chart for selecting universities and degree programme

The selection and review processes are presented in Fig. 1.

Frequency analyses were carried out, stratified by discipline and region (northern Europe, southern Europe, eastern Europe, and western Europe). In the latter case, Israel was excluded as it is not geographically in Europe. In addition, the Chi-square test was used to compare curriculum content by discipline (medicine vs. nursing) and European region. We considered p < 0.05 to be statistically significant.

## Results

Eighty-eight medical and nursing university websites were reviewed thoroughly. Included medical schools were in 7 northern European countries (Denmark, Estonia, Finland, Iceland, Ireland, Lithuania, and Sweden), 6 eastern European countries (Czech Republic, Moldova, Poland, Romania, Slovakia, and Turkey), 6 western European countries (Austria, Belgium, France, Germany, Netherlands, and Switzerland) and 7 southern European countries (Bosnia and Herzegovina, Italy, Malta, North Macedonia, Portugal, Serbia, and Spain). Nursing schools were in 7 northern European countries (Denmark, Finland, Iceland, Ireland, Lithuania, Norway, and Sweden), 3 eastern European countries (Poland, Slovakia, and Turkey), 5 western European countries (Austria, Belgium, Netherlands, Switzerland, and Germany), and 5 southern European countries (Croatia, Italy, North Macedonia, Portugal, and Spain), plus Israel, which is considered a cooperating member of the ERNST Consortium.

Table 1 shows the data on the included universities.

**Table 1:** Summary of universities and subjects studied

	N Countries	N universities	Public	Private	Public-private	Modules selected
Nursing	29	44	34	8	1	54
Medicine	29	44	41	3	0	48
Total	29*	88	75	12	1	102

* The same countries were reviewed for medicine and nursing schools, so N is 29 in total.

Among the contents analysed in nursing degrees, quality of care and interprofessional communication were the best covered topics, while no content was available on the second victim phenomenon. In nursing, some modules covered most of the contents studied (Table 2). For example, one module taught the following topics: “Systems thinking”; “Preventive systematic safety work”; “Safety culture”; “Teamwork; Communication”; “Regulations”; “Care injuries”; and “Risk of medical damage”.

In medicine degrees, the included topics were not as well covered as in nursing, with the exceptions of adverse events and second victims (Table 2). Among the contents analysed, interprofessional communication was the best covered topic. There was one medical school that taught content on second victims as part of a standalone module covering all patient safety issues, including: “Clinical safety: an essential dimension of the quality of care”; “Epidemiology and individual study of adverse effects, how to recognise adverse effects related to health care”; “What an error is: medical errors, Medication errors”; “Prevention of adverse effects”; “Risk management”; “Avoiding failures. It should not happen: a priori prevention of adverse effects”; “Avoiding failures. Why it happened: a posteriori prevention of adverse effects”; “Working safely: safe clinical practices and clinical alerts”; “Patient-centred care. Risk communication”. Practice seminars focused on: “The responsibility of the physician”, “Good practices in the care of second victims”, and “Clinical safety of the patient”.

**Table 2:** Patient safety in the nursing and medical curricula of COST Action universities

	Patient safety	Quality of care	Risk management	Safe practices	Interprofessional communication	Adverse events	Second victims
**Nursing**							
Northern Europe	8	9	2	2	15	1	0
Southern Europe	7	7	5	3	11	2	0
Western Europe	3	7	3	2	8	0	0
Eastern Europe	4	4	4	3	5	3	0
Total, % (n)*	52.4 (22)	64.3 (27)	33.3 (14)	23.8 (10)	92.9 (39)	14.3 (6)	0 (0)
Total, % (n)	50.0 (22)	65.9 (29)	31.8 (14)	22.7 (10)	93.2 (41)	13.6 (6)	0 (0)
**Medicine**							
Northern Europe	4	3	2	1	6	2	0
Southern Europe	4	5	1	1	9	2	1
Western Europe	6	4	0	0	12	2	0
Eastern Europe	2	5	1	1	7	2	0
Total, % (n)	36.4 (16)	38.6 (17)	9.1 (4)	6.8 (3)	77.3 (34)	18.2 (8)	2.3 (1)

*Excluding Israeli universities, N = 42

Table 3 shows the differences in patient safety and second victim topics between nursing and medicine and European regions. Significant differences were found between nursing and medicine curricula contents in quality of care (χ^2^ (1, 88) = 6.56, p = 0.01) risk management (χ^2^ (1, 88) = 6.98, p = 0.01), safe practices (χ^2^ (1, 88) = 4.42, p = 0.04), and interprofessional communication (χ^2^ (1, 88) = 4.42, p = 0.04). No differences were found between the curricula contents by European region.

**Table 3:** Differences in the content on patient safety, second victims and quality of care in curricula according to discipline (medicine vs. nursing) and European region (north, south, east, and west)

Topic	Nursing (n = 44)	Medicine (n = 44)	p-value^*^	NE (n = 23)	SE (n = 22)	EE (n = 17)	WE (n = 24)	p-value	
Patient safety	50.0 (22)	36.4 (16)	0.20	52.2 (12)	50.0 (11)	35.2 (6)	37.5 (9)	0.60	
Quality of care	65.9 (29)	38.6 (17)	0.01	52.2 (12)	54.5 (12)	52.9 (9)	45.8 (11)	0.94	
Risk management	31.8 (14)	9.1 (4)	0.01	17.4 (4)	27.3 (6)	29.4 (5)	12.5 (3)	0.48	
Safe practices	22.7 (10)	6.8 (3)	0.04	13.0 (3)	18.2 (4)	23.5 (4)	8.3 (2)	0.57	
Interprofessional communication	93.2 (41)	77.3 (34)	0.04	91.3 (21)	90.9 (20)	70.6 (12)	83.3 (20)	0.25	
Adverse events	13.6 (6)	18.2 (8)	0.56	13.0 (3)	18.2 (4)	29.4 (5)	8.3 (2)	0.32	
Second victims	0 (0)	2.3 (1)	-	0 (0)	4.5 (1)	0 (0)	0 (0)	-	

% (n)

^*^ Chi-square Test, level of significance p < 0.05

NE: Northern Europe; SE: Southern Europe; EE: Eastern Europe; WE: Western Europe

## Discussion

Our study shows that patient safety is still neglected in medicine and nursing curricula in European universities, representing a contradiction between the patient safety competencies that health professionals need in clinical practice and those that clinical curricula currently provide. Considering the frequency with which adverse events occur in hospitals and primary care [14] and the need to introduce elements such as healthcare risk management, transparency in reporting to patients who suffer an adverse event, or peer support to deal with highly stressful situations, there is a long way to go in adapting training programmes to patient safety needs. Of the universities analysed, half of the nursing schools and 60% of the medical schools – including all the medical schools in Croatia, North Macedonia, and Italy – did not cover any patient safety topics. Similarly, none of the nursing schools in Denmark, Estonia, and Malta included specific curricular content on patient safety topics.

Our results show significant differences between universities, reflecting the uneven implementation of patient safety topics in medical and nursing schools, despite the existence of guidelines such as the WHO’s [8]. Patient safety may be considered as very present in medical and nursing curricula due to the content related to interprofessional communication. However, if we understand patient safety as a transversal topic with relevance to all medical and nursing fields, it is clear that it is not taught in a comprehensive way. In both medicine and nursing, the shortcomings are centred on the teaching of risk management, safe practices, adverse events, and the impact of the second victim phenomenon. These results indicate that patient safety is not being covered with the necessary depth and breadth. Other studies have drawn similar conclusions from the results of a patient safety knowledge test [9], student self-reports on experience and competence [10], a survey of higher education institutions [11], and a documentary review [12].

In addition, curricula generally do not reflect key issues that directly involve the patient, such as open and honest discussion of adverse events resulting in harm (open disclosure), patient rights and fair compensation for harm suffered, and the active role of the patient as the second line of control. We found only two cases in medicine and one in nursing that addressed the communication of adverse events to the patient.

Some exceptional degree programmes, where patient safety was well integrated into the curricula, stood out, particularly the standalone module in one medical school and a less complete case in a nursing school. However, these examples were few and far between.

In Europe, medical and nursing schools have been slowly implementing curricula addressing patient safety. Nevertheless, the Patient Safety and Quality of Care Working Group highlighted that the education and training of health professionals was one of the least implemented areas of the European patient safety recommendations [15]. We also know that the development of learning environments in coordination between academic and healthcare settings achieves better results [16]. Furthermore, the absence of content in the curricula does not follow the course of action proposed in the WHO Global Action Plan for Patient Safety 2021–2030 [1]. That document recommends incorporating patient safety into both university curricula and continuing professional development, with an emphasis on interprofessional learning. Given the importance and nature of patient safety, training in this area should ideally begin in the early years to enable students to acquire the competencies needed for clinical practice at a lower cost than some years later during continuous professional training. Studies have indicated that students need competencies both to prevent errors and to act safely after an error has occurred [17]. During their training, students commonly witness the occurrence of safety events and unsafe practices [18], but they are unable to “speak up” during a critical situation in 56% of cases, even when they can do so [19]. Universities, and particularly student mentors, must transmit transferable skills to deal with such events [20].

The outcomes of various educational initiatives worldwide prove that patient safety education improves students’ knowledge, skills, and attitudes about safety, raising their awareness of the importance of addressing medical errors in their future careers [21–26]. That said, initiatives focused on improving student training in patient safety should be accompanied by assessment. Several scales have been developed to assess what students know and what information they receive to model a culture of clinical safety [27–29]. These scales have been useful for evidencing training needs, but they can also be useful for assessing the effectiveness of educational initiatives aimed at improving students’ knowledge and competencies.

It is essential to address the difficulties that hinder patient safety from being implemented in university curricula. Human factors, resource shortages, and challenges in changing existing structures help explain this challenge [1]. Specific factors include a lack of familiarity with patient safety among instructors and educators; reluctance of institutions to teach knowledge outside clinical disciplines because curricula are already complete; rigidity of the curricula themselves to incorporate new areas; weaknesses in educational coordination and planning; shortages in time, funding, or skills; and limited recognition and interest among educators with the capacity to introduce curricular changes [7, 30, 31].

The lack of training in patient safety can result in avoidable adverse events and medical errors, which directly affects patients but can also make professionals second victims. Recognition of this phenomenon and its implications for patient safety will affect healthcare organisations’ ability to reduce the incidence of similar adverse events [8, 32–34]. Students are also vulnerable to becoming second victims as soon as they begin their first clinical experiences [35, 36]. Several studies report that at this stage, up to 75% of students witness adverse events [37, 38], and 25% were directly involved in them [39]. Another study noted that more than 80% of students who were involved in a significant adverse event at some point in their clinical practice suffered second victim symptoms [36]. These topics must therefore also be contemplated in educational programmes so that students are prepared to deal with the occurrence of adverse events in their professional careers.

## Conclusion

Our study showed that patient safety remains neglected in the medical and nursing curricula of European universities. Specifically, the phenomenon of the second victim was only found to be integrated in the curricula of one of the universities. However, positive initiatives were also found. Adverse events occur frequently in hospitals and primary care, so it is necessary for healthcare professionals to have the necessary patient safety competencies to deal with these situations. To this end, further work is needed to introduce patient safety elements, such as healthcare risk management, transparency in the information provided to patients who suffer an adverse event or the second victim phenomenon, into medical and nursing training.

## Limitations

The data analysed is limited to publicly available information on university websites. This way, the willingness to randomise the process of selection of universities was limited by access to information. Also, the difference in the number of universities available in the different countries could lead to a possible bias in the representation of countries in the final sample. Although an attempt was made to adapt the exclusion criteria for countries with a smaller number of universities in order to have an adequate sample from these countries, an over-representation may have occurred, which should be taken into account in the conclusions of this study. In addition, it should be noted that in order to review the university websites, where there was no English version, machine translator of the national language was used, so the translations may not be entirely accurate. Despite all these limitations both the number of academic centres considered, their scope, and the random selection allow us to consider that the results of this study reflect the current situation of educational programs for the new generations of physicians and nurses in Europe.

The potential complementary training activities offered by universities, hospitals during the residency training period, and other sources of training, such as professional associations, were not included. All the curricula reviewed, both in nursing and medicine, include these stages of practice in healthcare centres, so that, although the curricula do not formally include patient safety content, it may be formally or informally present in these clinical settings. In addition, the information published in the curricula may not reflect all the training activities carried out. The gaps reflected in this study may therefore be corrected in practice.

However, the training regulated by curricula reflects a need to rethink what is taught in medical and nursing schools across Europe. We also need to consider that some patient safety issues might be integrated into the different clinical learning modules. In this case, the description of the module may not be available, but patient safety topics could be discussed (e.g. hospital infections in the module on communicable diseases, or prevention of surgical adverse events in the module on surgery). Future studies should focus on the learning outcomes of the curricula and modules to get a more complete overview.

### Electronic supplementary material

Below is the link to the electronic supplementary material.

Supplementary Material 1

## Data Availability

The datasets generated and analysed during the current study are available in the OSF repository, https://osf.io/zur5q/.
