# Supplementary material for: Patient safety topics, especially the second victim phenomenon, are neglected in undergraduate medical and nursing curricula in Europe: an online observational study
Source: BMC Nurs. 2023 Aug 24;22:283. doi: 10.1186/s12912-023-01448-w (PMC10464449; doi:10.1186/s12912-023-01448-w)
Supplement: Supplementary file 1 — Supplementary Material 1 [file 12912_2023_1448_MOESM1_ESM.docx]

Additional file 1. List of included and excluded universities and curricula

Supplementary table 1. Medical universities and curricula excluded (n=74)

| Country | Medical schools excluded |
| --- | --- |
| AUSTRIA | 1. University of Linz |
| BELGIUM | 1. Free University of Brussels |
|  | 1. University of Antwerp |
|  | 1. Catholic University of Louvain (UCLouvain) |
| BOSNIA AND HERZEGOVINA | 1. University of Banja Luka |
| CROATIA | 1. University of Rijeka |
|  | 1. Catholic University of Croatia |
|  | 1. University of Zagreb |
| CZECH REPUBLIC | 1. Charles University |
| DENMARK | 1. University of Copenhagen |
| FINLAND | 1. University of Oulu |
| FRANCE | 1. University of Nantes |
|  | 1. University of Burgundy |
|  | 1. Clermont Auvergne University |
|  | 1. University of Caen Normandy |
|  | 1. University of Rennes 1 |
| GERMANY | 1. University of Tübingen |
|  | 1. Free University of Berlin |
|  | 1. University of Göttingen |
|  | 1. University of Freiburg |
|  | 1. University of Hamburg |
| IRELAND | 1. Royal College of Surgeons in Ireland |
| ISRAEL | 1. Bar-Ilan University |
|  | 1. Zefat Academic College |
| ITALY | 1. University of Bologna |
|  | 1. University of Padua |
|  | 1. University of Milan |
|  | 1. University of Milano-Bicocca |
|  | 1. University of Turin |
|  | 1. University of Naples Federico II |
|  | 1. Vita-Salute San Raffaele University |
|  | 1. University of Pisa |
|  | 1. Tor Vergata University of Rome |
|  | 1. University of Genoa |
|  | 1. University of Verona |
| LITHUANIA | 1. Vilnius University |
|  | 1. Lithuanian University of Health Sciences |
| NETHERLANDS | 1. Radboud University |
|  | 1. University of Groningen |
|  | 1. VU Amsterdam - Vrije Universiteit Amsterdam |
|  | 1. Maastricht University |
| NORTH MACEDONIA | 1. Goce Delcev University of Stip |
|  | 1. State University of Tetova |
| NORWAY | 1. University of Oslo |
|  | 1. University of Bergen |
| POLAND | 1. Poznan University of Medical Sciences |
| PORTUGAL | 1. NOVA University Lisbon |
|  | 1. Catholic University of Portugal |
| ROMANIA | 1. Transilvania University of Brașov |
|  | 1. University of Medicine, Pharmacy, Science and Technology of Târgu-Mureş |
|  | 1. University of Medicine and Pharmacy, Timișoara |
|  | 1. University of Medicine and Pharmacy of Craiova |
|  | 1. Iuliu Haţieganu University of Medicine and Pharmacy of Cluj-Napoca |
|  | 1. Titu Maiorescu University |
| SLOVAKIA | 1. Slovak Medical University in Bratislava |
|  | 1. Pavol Jozef Šafárik University |
| SPAIN | 1. University of Barcelona |
|  | 1. Complutense University of Madrid |
|  | 1. Jaume I University |
|  | 1. University of Cádiz |
| SWEDEN | 1. Uppsala University |
|  | 1. Karolinska Institute |
|  | 1. Örebro University |
| SWITZERLAND | 1. Swiss Federal Institute of Technology in Zürich |
|  | 1. University of Geneva |
|  | 1. University of Bern |
|  | 1. University of Italian Switzerland/ Università della Svizzera Italiana |
|  | 1. University of Fribourg |
| TURKEY | 1. Gazi University |
|  | 1. Istanbul Medeniyet University |
|  | 1. Atatürk University |
|  | 1. Selçuk University |
|  | 1. Recep Tayyip Erdoğan University |
|  | 1. Karamanoğlu Mehmetbey University |

Supplementary table 2. Medical universities and curricula included according to the inclusion criteria (n=44)

| Country | Medical schools included |
| --- | --- |
| AUSTRIA | 1. Medical University of Graz |
|  | 1. Medical University of Vienna |
|  | 1. Medical University of Innsbruck |
| BELGIUM | 1. KU Leuven |
|  | 1. Ghent University |
| BOSNIA AND HERZEGOVINA | 1. University of Zenica |
| CZECH REPUBLIC | 1. Masaryk University |
|  | 1. Palacký University Olomouc |
| DENMARK | 1. Aarhus University |
|  | 1. University of Southern Denmark |
| ESTONIA | 1. University of Tartu |
| FINLAND | 1. University of Turku |
| FRANCE | 1. Claude Bernard University Lyon 1 |
| GERMANY | 1. Technical University of Munich |
|  | 1. RWTH Aachen University |
| ICELAND | 1. University of Iceland |
| IRELAND | 1. Trinity College Dublin |
|  | 1. University College Cork |
| MALTA | 1. University of Malta |
| MOLDOVA | 1. Nicolae Testemițanu State University of Medicine and Pharmacy |
| NETHERLANDS | 1. University of Amsterdam |
|  | 1. Leiden University |
|  | 1. Erasmus University Rotterdam |
| POLAND | 1. Jagiellonian University |
|  | 1. Medical University of Łódź |
| PORTUGAL | 1. University of Porto |
|  | 1. University of Lisbon |
| ROMANIA | 1. Carol Davila University of Medicine and Pharmacy |
| SERBIA | 1. University of Kragujevac |
| SLOVAKIA | 1. Comenius University in Bratislava |
| SPAIN | 1. University of Almería |
|  | 1. Autonomous University of Madrid |
|  | 1. University of Valencia |
|  | 1. University of Zaragoza |
|  | 1. Miguel Hernández University of Elche |
| SWEDEN | 1. University of Gothenburg |
| SWITZERLAND | 1. University of Basel |
|  | 1. University of Lausanne |
|  | 1. University of Neuchâtel |
| TURKEY | 1. Hacettepe University |
|  | 1. Istanbul University |
|  | 1. Ankara University |
|  | 1. Ege University |
|  | 1. Yeditepe University |

Supplementary table 3. Nursing universities and curricula excluded (n=44)

| Country | Nursing schools excluded |
| --- | --- |
| AUSTRIA | 1. Medical University of Graz |
|  | 1. UMIT Tirol – Private University for Health Sciences and Health Technology |
| BELGIUM | 1. Karel de Grote University of Applied Sciences and Arts |
| CROATIA | 1. University of Rijeka |
|  | 1. University of Dubrovnik |
| CZECH REPUBLIC | 1. Charles University |
| DENMARK | 1. UCL University College |
|  | 1. University College South Denmark |
| ESTONIA | 1. Tartu Health Care College |
| FINLAND | 1. University of Tampere |
|  | 1. Arcada University of Applied Sciences |
| FRANCE | 1. Paris Cité University |
|  | 1. University of Versailles Saint-Quentin-en-Yvelines |
|  | 1. University of Rennes 1 |
| IRELAND | 1. University of Galway |
| ISRAEL | 1. Zefat Academic College |
|  | 1. Jerusalem College of Technology - Lev Academic Center |
| ITALY | 1. Sapienza University of Rome |
|  | 1. University of Bologna |
|  | 1. University of Milano-Bicocca |
|  | 1. University of Turin |
|  | 1. University of Naples Federico II |
|  | 1. University of Pisa |
|  | 1. Tor Vergata University of Rome |
|  | 1. University of Verona |
| LITHUANIA | 1. Klaipėda State College |
| MALTA | 1. University of Malta |
| NORTH MACEDONIA | 1. State University of Tetova |
| NORWAY | 1. University College of South-Eastern Norway |
|  | 1. University of Agder |
| POLAND | 1. Medical University of Warsaw |
|  | 1. Poznan University of Medical Sciences |
|  | 1. Medical University of Białystok |
| PORTUGAL | 1. Nursing School of Porto |
| ROMANIA | 1. University of Medicine and Pharmacy of Craiova |
| SPAIN | 1. University of Barcelona |
|  | 1. University of Valencia |
| SWEDEN | 1. Karolinska Institute |
|  | 1. Malmö University |
|  | 1. Linnaeus University |
|  | 1. Dalarna University College |
| TURKEY | 1. Atatürk University |
|  | 1. Istanbul Bilgi University |
|  | 1. Recep Tayyip Erdoğan University |

Supplementary table 4. Nursing universities and curricula included according to the inclusion criteria (n=44)

| Country | Nursing schools included |
| --- | --- |
| AUSTRIA | 1. University of Applied Sciences Joanneum |
|  | 1. Salzburg University of Applied Sciences |
|  | 1. Carinthia University of Applied Sciences |
| BELGIUM | 1. KU Leuven |
| CROATIA | 1. University of Zadar |
|  | 1. ZVU University of Applied Health Sciences |
| DENMARK | 1. University College Copenhagen |
| FINLAND | 1. Satakunta University of Applied Sciences |
| FRANCE | 1. University of Angers |
| GERMANY | 1. University of Freiburg |
| ICELAND | 1. University of Iceland |
| IRELAND | 1. University College Cork |
|  | 1. University of Limerick |
| ISRAEL | 1. The Academic College of Tel Aviv-Yaffo |
|  | 1. Ashkelon Academic College |
| ITALY | 1. Vita-Salute San Raffaele University |
|  | 1. University of Genoa |
| LITHUANIA | 1. Alytaus Kolegija/ University of Applied Sciences |
|  | 1. Utena College |
| NETHERLANDS | 1. NCOI University of Applied Sciences |
|  | 1. Avans University of Applied Sciences |
| NORTH MACEDONIA | 1. Goce Delcev University of Stip |
|  | 1. South East European University |
| NORWAY | 1. Norwegian University of Science and Technology |
|  | 1. University of Stavanger |
|  | 1. VID Specialized University |
| POLAND | 1. Jagiellonian University |
| PORTUGAL | 1. ESEnfC Escola Superior de Enfermagem de Coimbra (Higher School of Nursing) |
| SLOVAKIA | 1. University of Prešov |
| SPAIN | 1. University of Almería |
|  | 1. Complutense University of Madrid |
|  | 1. Autonomous University of Madrid |
|  | 1. University of Zaragoza |
|  | 1. University of Cádiz |
| SWEDEN | 1. Uppsala University |
|  | 1. University of Gothenburg |
|  | 1. Linköping University |
|  | 1. Örebro University |
|  | 1. Jönköping University |
| SWITZERLAND | 1. Kalaidos University of Applied Science |
|  | 1. University of Applied Sciences and Arts of Southern Switzerland |
| TURKEY | 1. Ankara University |
|  | 1. Ege University |
|  | 1. Istanbul Medeniyet University |
